# Recent Advances in Flexible Tactile Sensors for Intelligent Systems

**DOI:** 10.3390/s21165392

**Published:** 2021-08-10

**Authors:** Yiyao Peng, Ning Yang, Qian Xu, Yang Dai, Zhiqiang Wang

**Affiliations:** Information Science Academy of China Electronics Technology Group Corporation, Beijing 100086, China; pengyiyao924@163.com (Y.P.); yangning8848@163.com (N.Y.); xuqian199004@163.com (Q.X.); daiyang_2000@sohu.com (Y.D.)

**Keywords:** tactile sensor, flexible sensing, multifunction, intelligent system

## Abstract

Tactile sensors are an important medium for artificial intelligence systems to perceive their external environment. With the rapid development of smart robots, wearable devices, and human-computer interaction interfaces, flexible tactile sensing has attracted extensive attention. An overview of the recent development in high-performance tactile sensors used for smart systems is introduced. The main transduction mechanisms of flexible tactile sensors including piezoresistive, capacitive, piezoelectric, and triboelectric sensors are discussed in detail. The development status of flexible tactile sensors with high resolution, high sensitive, self-powered, and visual capabilities are focused on. Then, for intelligent systems, the wide application prospects of flexible tactile sensors in the fields of wearable electronics, intelligent robots, human-computer interaction interfaces, and implantable electronics are systematically discussed. Finally, the future prospects of flexible tactile sensors for intelligent systems are proposed.

## 1. Introduction

With the development of science and technology, mankind is entering the era of intelligence [1]. Mobile internet and smart terminals have developed rapidly in recent years, which greatly stimulated the exploration of smart sensing technologies in the fields of human-computer interaction, artificial intelligence, and wearable devices [2,3,4,5,6,7]. Traditional pressure sensors are usually made of rigid materials, greatly limiting their applications in flexible contacts or wearable devices [8,9]. Therefore, in the past ten years, a variety of flexible sensors and electronic skins that mimic the tactile functions of human skin have been developed, which can also quantify the sensations such as force, temperature, and humidity [10,11,12,13,14,15]. Flexible tactile sensors are flexible electronic devices that convert tactile signals into electrical signals. They have huge application prospects in the fields of smart systems such as wearable electronic equipment, health and sports monitoring, biomedicine, software robots, and human-computer interactions [16].

According to working mechanisms, tactile sensors are mainly based on piezoresistive effects, capacitive effects, piezoelectric effects and triboelectric effects [17,18]. Among them, piezoresistive tactile sensor has the merits of high frequency response, simple signal processing, simple structure, and low cost, and whose resistivity changes with external pressure stimuli [19]. The main limitations of piezoresistive sensors are poor repeatability, sensitive to temperature, and hysteresis [10]. The capacitance change for capacitive tactile sensors is influenced by the magnitude of the external pressure and the relative position between the parallel plates. The advantages for capacitive sensors are high sensitivity, low power consumption, and large area integration, and the main disadvantages can be concluded as parasitic capacitance, sensitivity to noise and complex measurement circuits [20]. The piezoelectric tactile sensor is based on the polarization of the piezoelectric material induced by applied strain results in the change of electric potential, with characteristics including high natural frequency, high sensitivity, and stable performance [9]. However, it is not applicable to measure static pressure signal but only dynamic pressure signal [21]. With regard to the triboelectric tactile sensors, it is mainly based on the frictional electrification and electrostatic induction effects to affect the flow of electric charge, has superiority in terms of high instantaneous power, and is self-powered. The signal interference generated by external electrostatic induction is an important problem to be solved in the process of its application [22]. In addition to the transduction mechanisms mentioned above, the four types of flexible tactile sensors, and other transduction principles such as optical and resonant tactile sensing, have also been reported to sense pressure [23,24].

At present, flexible tactile sensors are profoundly changing the lives of human beings. Flexible and even stretchable devices are becoming closer to human skin and expand the application range of tactile sensors [23,25,26]. The development of flexible tactile sensor devices has mainly unfolded from two orientations, namely high performance and multifunction perspectives. High performance refers to the notion that flexible sensor devices are developing in continuous improvement of performance parameters, including high resolution, high sensitivity, fast response time, detection range, etc. In order to meet practical applications, single-function tactile sensors are developing towards multifunction use in terms of being self-powered, visualization capabilities, biodegradability, being self-healing, etc. [27,28,29,30,31,32]. Among them, we focus on the recent representative development of high-resolution, high-sensitivity, self-powered, and visualization capabilities which are also the key development direction of flexible tactile sensing in the future. In the researches of high-resolution tactile sensing, through the introduction of piezoelectric nanomaterial and piezoelectric and piezo-phototronic effects, electronic skin now has a resolution of up to 6350 dpi, exceeding that of human skin and further promoting the development of intelligent robots and human-computer interaction interfaces [9]. Ultrahigh sensitive tactile sensors have higher accuracy and lower detection limit, and the pressure of 3 Pa can be detected [33]. Self-powered tactile sensors can collect or convert natural energy [34]. Since the discovery of nanogenerators by Wang Group in 2006, self-powered flexible tactile sensors have been regarded as promising candidates in the field of smart electronics, which provide an effective way to develop low power consumption, long life spans, and environmental-friendly tactile sensors [35,36,37,38]. In addition to sensing, user interaction functions are also very attractive for future electronic skins [39]. By integrating tactile pressure-sensitive sensors with organic light-emitting diodes, or based on changes in the refractive index of the piezoelectric nanomaterial resonant cavity, multifunctional visual sensor devices can be realized [40,41,42,43]. The applications of high-performance and multifunctional flexible tactile sensors mainly include wearable electronics, smart robots, human-computer interaction interfaces and implantable electronics, which can be employed in the field of human health diagnosing, environmental monitoring, prosthetics, electronic signatures, and biomedical therapies [44,45,46]. In order to better adapt to these emerging applications, achieving quantitative measurement, strong stability and robustness, and multifunction capabilities still face huge challenges [47].

This review mainly introduces the recent progress on flexible tactile sensors for intelligent systems. Firstly, the main transduction mechanisms of tactile sensing are summarized. And then, the recent progress of high-performance and multifunctional flexible tactile sensors are discussed, mainly focus on high resolution, high sensitivity, self-power, and visualization. On this basis, the potential applications of high-performance flexible tactile sensing in smart systems such as wearable electronics, smart robots, human-computer interaction interfaces, and implantable electronics are introduced. Finally, the challenges in flexible tactile sensing for practical applications and future trends in development are proposed.

## 2. Transduction Mechanisms

**Figure 1:**
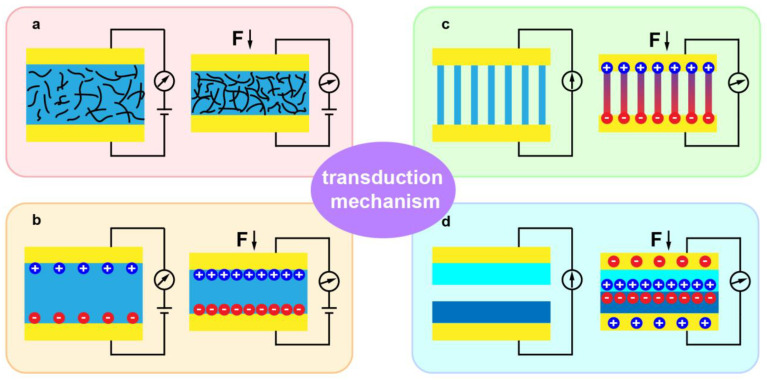
Schematic illustrations of the four typical transduction mechanisms: (**a**) piezoresistive, (**b**) capacitive, (**c**) piezoelectric, and (**d**) triboelectric sensing.

The tactile sensing abilities of electronic skin could be summarized as the perception of strain, pressure, shear force, twist deformation, etc. Generally, the common transduction methods that covert mechanical stimulation into electrical signals can be sum up as piezoresistivity, capacitance, piezoelectricity, and triboelectricity [18]. Figure 1 presents the four types of transduction mechanisms for flexible tactile sensors.

### 2.1. Piezoresistive Tactile Sensors

The principle of the piezoresistive tactile sensor is based on the piezoresistive effect, that is, the resistance of the materials changes under the applied external mechanical stimuli [48]. (Figure 1a) Piezoresistive tactile sensors have attracted lots of attentions due to their high sensitivity, simple structure, and low cost. With the development of piezoresistive tactile sensors technology, piezoresistive crystals, strain gauges, and composite piezoresistive materials have been concluded as the three different piezoresistive sensing types [49].

Piezoresistive crystals usually include silicon or other semiconductors. Various semiconductors with inherent piezoresistive effects, such as Si, CNT, graphene, α-In_2_Se_3_, MoS_2_, VO_2_, and PtSe_2_, are introduced into piezoresistive tactile sensors to realize tactile sensing via band structure changes under external strain [50,51,52,53,54]. Although the piezoresistive crystal possesses brittle and rigid characteristics, it can be integrated on a flexible substrate, such as polyimide materials, so as to realize flexible tactile sensing, miniaturization, and high-density integration [55]. Similar to piezoresistive crystals, strain gauge-based tactile sensors are made of metal materials, whose resistance value is directly related to the volume change [19]. Metal materials, including metal (Au, Ag, Pt, Al, ITO, etc.) nanowires, nanoparticles, and thin film, possess high conductivity compared with other materials, and could be employed as an active layer for flexible tactile sensors [56,57]. Elastic materials are generally employed as the substrates to metal strain gauge sensors, thus, the resistance of the tactile sensor change correspondingly when the substrate is deformed [58]. Recently, piezoresistive composites, usually formed by embedding conductive fillers into insulating organic materials, have been widely researched owing to excellent flexibility and adjusted pressure sensitivity [59]. The types of conductive fillers are abundant, from zero-dimensional nanoparticles (NPs), such as carbon black, Au NPs, and Ag NPs, to one-dimensional NWs or nanotubes, such as Ag NWs, Au NWs, and CNTs, to two-dimensional nanosheets, such as graphene, Ag flakes and Mxene (Ti_3_C_2_). Commonly used polymer matrices include PDMS, Ecoflex, PU, PVA, hydrogels and so on [10,47,60,61,62,63]. The resistance variation of piezoresistive composites can be attributed to the facture and regeneration of conductive path caused by the filler inside materials under an applied pressure. Among them, composite materials are considered the most promising and also the most recently explored piezoresistive materials for applications in tactile sensors due to a high sensitivity and cycling stability. On the other hand, conductive polymeric composites possess more choices in materials selection and structure design. The Bao group has done a lot of enlightening and creative research work in this respect [11].

### 2.2. Capacitive Tactile Sensors

Capacitance (C) stands for the capability of a capacitor to store charges. Figure 1b presents a general capacitor construction with a dielectric sandwiched by two parallel plates. C = εA/d is used to describe capacitance, where ε represents the dielectric constant, and A and d stand for the overlap area and distance between the two plates, respectively. Typically, the different types of force and strain, such as pressure or shear force, can be detected through changing d or A [17]. It has been demonstrated that capacitive tactile sensing possesses superior sensitivity, static force measurement, and low power consumption. The most direct method of capacitive tactile sensor is to use a metal film as an electrode and an elastomer as a dielectric layer sandwiched between two electrodes. In order to improve the mechanical properties of the metal film during deformation, the network electrode structure made of CNTs or metal NWs has been widely studied and applied [24,64]. To further enhance the sensitivity of the sensor, the middle elastomer dielectric can choose PDMS or Ecoflex with micro/nano structure, such as pyramids, microlines, microrods, and hemispheres. At the same time, other functions such as self-repair and biodegradation of capacitive tactile sensors are also realized via new materials such as poly(glycerol sebacate) [65]. Bao et al. reported capacitance-dependent flexible sensor array for highly sensitive touch perception based on carbon nanotube electrodes on flexible rubber substrate layer [66]. With the rapid development of flexible field effect transistors, capacitive sensors with variable effective permittivity have attracted great interest in tactile sensing. A novel organic field effect transistor based on microstructured PDMS gate dielectric layer is proposed, which also has been fabricated into a tactile sensor array with an extremely great sensitive pressure sensing [33,67].

### 2.3. Piezoelectric Tactile Sensors

Piezoelectric polarization occurs inside some dielectrics under an external force along a certain direction, which will result in the occurrence of electrical dipole moments and induce positive and negative charges appear on their two opposite surfaces, as shown in Figure 1c [68]. The polarization charge density is positively correlated to mechanical force. This phenomenon is called the piezoelectric effect, generally generating in oriented non-centrosymmetric crystal structures, and the piezoelectric coefficient is an important parameter to measure piezoelectric performance [69]. Piezoelectric polymers and inorganic piezoelectric materials are commonly used in the construction of piezoelectric tactile sensors. Current advanced tactile sensors based on piezoelectric polymers mainly rely on thin-film geometry, with PVDF and its derivatives as a typical example [70,71]. Due to the low piezoelectric constant of piezoelectric polymers restricts the realization of high-performance tactile sensors, inorganic piezoelectric materials have attracted more attention, such as ZnO, PZT, GaN, CdS, and some two-dimensional materials with asymmetric centers [72,73,74,75]. The material structure includes nanobelts, nanofibers, nanowires, nanosheets and nanospheres. The common material of ZnO is the typical piezoelectric semiconductors owing to the lack of centrosymmetry. The Wang group reports lots of research works on piezotronic devices to realize high-resolution tactile sensor imaging, since the piezoelectric potential generated in the material could regulate the Schottky barrier height under an applied pressure [76,77,78]. Additionally, the piezoelectric organic compounds represented bypolyvinylidene fluoride (PVDF) not only have good piezoelectric properties, but also possess high flexibility and low density, and can therefore also be greatly utilized in the development of flexible tactile sensors [71].

### 2.4. Triboelectric Tactile Sensors

The triboelectric effect can be often observed in our life. When two different materials are in contact with each other via an external force, the surfaces of the two materials will generate positive and negative electrostatic charges, respectively [79]. Then, when the two contact surfaces are separated after releasing the force, the electrostatic charges will also be separated to induce a potential difference. If an external wire or load is connected between these two materials to form a loop, current will be generated due to electrostatic induction, as shown in Figure 1d. Nowadays, a triboelectric nanogenerator (TENG) has been put forward by the Wang group and has been widely studied, and is based on the coupling of triboelectric phenomenon and electrostatic induction [80,81]. The selection of commonly used materials for triboelectric tactile sensors can refer to the electron affinity ranking diagram. For example, PDMS and Teflon are excellent electronic materials, while silk and metal (Al, Ag, etc.) are common electronic materials [30]. Recently, various functional materials, such as hydrogels, ionic liquids, liquid metals, and conductive composite materials, have also been used in triboelectric tactile sensors to achieve such various functional characteristics [82,83,84]. In terms of structural design, triboelectric materials using microstructures or nanowires can effectively enhance the triboelectric effect between friction materials. The tactile sensors based on TENG have the characteristics of simple structure, high instantaneous power, and free power supply, and thus can be particularly utilized in the self-powered tactile sensing field [30].

## 3. Performances of Flexible Tactile Sensors

At present, major breakthroughs have been made in the research of flexible tactile sensors to enhance the performances of flexible tactile sensors. Table 1 summarizes materials and structure, sensitivity, and other performance parameters of some flexible tactile sensors. The device development of flexible tactile sensor unfolded from two orientations: high performance and multifunction. For the flexible tactile sensor itself, the essential performances of high sensitivity, high resolution, fast response time, and wide detection range, etc., are the vital factors for guaranteeing the ability to sense tiny pressure precisely and produce a high-resolution mapping matrix. On the other hand, in order to apply the flexible tactile sensor into extensive practical applications, the multifunction, such as self-powered, visualization, biocompatibility, and self-healing, are considered as the significant factors. In this section, we display the recent development of representative high-resolution, high sensitivity, self-powered, and visualization to enhance the comprehensive performance, which also point out the key development direction of flexible tactile sensing in the future. Additionally, other performances and functions of flexible tactile sensors have also been discussed.

### 3.1. High-Resolution Tactile Sensing

**Figure 2:**
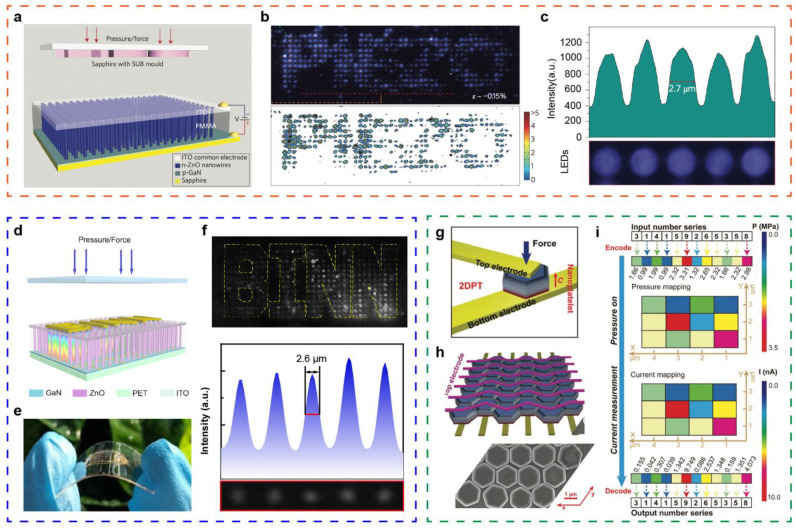
High-resolution flexible tactile sensors. (**a**) The structure diagram of a p-GaN/n-ZnO-based LED nanowires array applied in pressure sensor. (**b**) The distribution of the device under an applied pressure with a mould “piezo”. (**c**) Five typical nanowire LEDs and corresponding illumination intensity profile, estimating the resolution of 2.7 μm. Reproduced with permission from Ref. [9]. Copyright 2013, Springer Nature. (**d**) Schematic diagram of pressure distribution mapping though the flexible LED array sensor. (**e**) The physical image of the flexible sensor. (**f**) Detected “BINN” pressure distribution mapping of the flexible pressure sensor and corresponding five typical nanowire LEDs. Reproduced with permission from Ref. [88]. Copyright 2018, Elsevier Ltd. (**g**) Schematic illustration of two-terminal 2D piezotronic transistor based on ZnO nanoplatelet. (**h**) Images of a 2D piezotronic transistor array. (**i**) The distribution mapping from 3 × 4 tactile sensing array and corresponding electrical signal. Reproduced with permission from Ref. [89]. Copyright 2017, Wiley-VCH.

High-resolution flexible electronic devices promote the advance of robot technology, human-machine interfaces, and wearable electronics. Tactile sensing devices with high resolution could identify the position, direction, and appearance of complex objects more accurately [27,28,85]. Therefore, this performance of flexible tactile sensors is significant, and can be applied in smart signatures, electronic skins, and other directions. As discussed above, tactile sensors can be roughly divided into piezoresistive, capacitive, piezoelectric, and triboelectric. Tactile sensors based on piezoresistive or capacitive have been widely investigated, however, their resolution is generally in the millimeter level, not comparable to micrometer of human skin [86,87]. As an important type of tactile perception, piezoelectric-based tactile nanosensors can convert the external pressure into the internal polarization of the nanostructured material, achieving a higher resolution level, as shown in Figure 2. 

Pan et al. reported a high-resolution pressure sensor array based on nanowire LED composed of n-ZnO/p-GaN heterostructure in 2013, as shown in Figure 2a [9]. The emission intensity of nanowire LED under compressive strain would be enhanced based on the piezo-phototronic effect. When applying pressure with a “piezo” mold seal, a corresponding pressure distribution mapping can be collected clearly (Figure 2b). Five typical nanowire LEDs were selected, showing a high resolution of 2.7 μm (6350 dpi), as indicated in Figure 2c. However, the rigid sapphire substrate used in this work limits its application in intelligent skin and other fields due to the lack of flexibility. Subsequently, a series of flexible tactile sensors based on nanowire LEDs array were constructed, such as ZnO/PEDOT: PSS, CdS/PEDOT: PSS structures, and others, enduring a resolution decrease [72,74,90,91]. To realize the high-resolution two-dimensional pressure distribution image, a flexible LED array-based tactile sensor was fabricated by combining GaN film laser lift-off technology and hydrothermal epitaxial growth of patterned ZnO nanowires (Figure 2d) [88]. The physical image of the flexible sensor is shown in Figure 2e. When a convex mold is employed to apply pressure on the device, the luminescence intensity of local nanowires LED region under compressive strain was enhanced based on the piezo-phototronic effect, and the illumination intensity increases with the applied pressure, as displayed in Figure 2f. Thus, high-resolution pressure distribution mapping was generated, possessing a resolution up to 2.6 μm and a response time no less than 180 ms. Furthermore, a 2D piezotronic transistor matrix was fabricated by assembling a ZnO nanoplates chip array with a sandwich structure (Figure 2g,h) [89]. This device shows an excellent resolution of 12,700 dpi, exhibiting vital potential applications in adaptive high-resolution tactile sensors (Figure 2i). These works may be an important step in the high-resolution tactile sensing, which is expected to be applied in electronic skin, personalized signature, biological imaging, and optical MEMS.

### 3.2. Highly Sensitive Tactile Sensing

**Figure 3:**
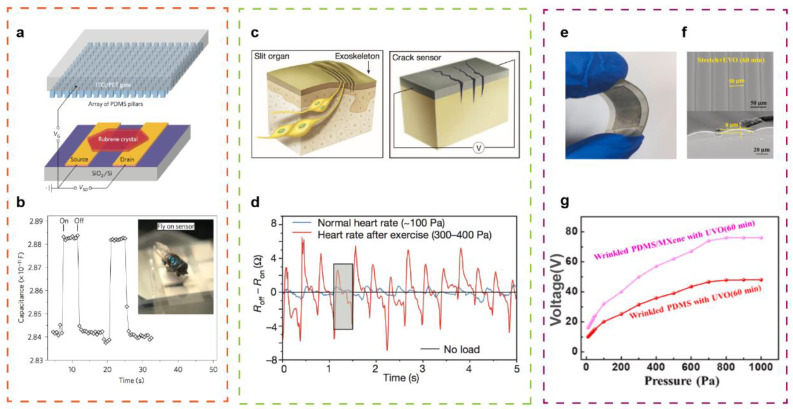
Highly sensitive flexible tactile sensors. (**a**) Layout of the capacitance- dependent tactile sensing organic transistors. (**b**) A highly sensitive flexible tactile sensor with an ultralow pressure sensing of 3 Pa. Reproduced with permission from Ref. [33]. Copyright 2010, Springer Nature. (**c**) Schematic illustrations of crack sensor inspired by the spider tactile sensing system. (**d**) The flexible tactile sensor for human physiology monitoring. Reproduced with permission from Ref. [92]. Copyright 2014, Springer Nature. (**e**) The optical image of the sensitive and self-powered flexible tactile sensor. (**f**) SEM image of PDMS after UVO irradiation. (**g**) A better performance of wrinkled PDMS/MXene compared with PDMS. Reproduced with permission from Ref. [96]. Copyright 2021, Elsevier Ltd.

In order to develop the touch perception comparable to human skin, flexible and even stretchable pressure-sensitive tactile sensors are crucial. Bao and co-workers fabricated a novel organic field-effect transistor based on microstructured PDMS gate dielectric layer with a highly sensitive pressure sensing, as presented in Figure 3a [33]. Compared with unstructured and other microstructured rubber, it has been demonstrated that the pyramid-structured PDMS film will contribute to enhance the pressure sensitivity of tactile sensors. According to the field-effect transistor theory, drain/source current is greatly influenced by applied external mechanical stimuli, since drain/source current is proportional to the specific gate capacitance. The flexible device can sense an extremely low pressure of 3 Pa, presenting an ideal choice for e-skin applications. (Figure 3b)

To further investigate the multi-functional flexible tactile sensor with ultra-high sensitivity and durability simultaneously, a nanoscale crack-based sensor made of Pt film and polymer polyurethane acrylate has been developed and reported, inspired by the slit organ of spider legs that can perceive small mechanical stress changes [92]. (Figure 3c,d).This flexible tactile sensor has been proven to be able to flexibly adhere to human skin to monitor human physiological signals and recognize voice patterns. Furthermore, it can also be applied in other ultra-sensitive tactile detection, with the gauge factor exceeds 2000 in the strain range of 0–2%.

The flexible tactile sensors on account of triboelectric transmission can produce self-powered and high-sensitive characteristics. A flexible triboelectric tactile sensor composed by wrinkled PDMS/MXene composite films under UVO irradiation is prepared, showing an ultrahigh sensitivity (Figure 3e,f) [96]. The optimal sensitivity is 0.18 V/Pa at the range of 10~80 Pa and 0.06 V/Pa between 80 Pa and 800 Pa, better than most reported self-powered tactile sensors (Figure 3g). The self-powered and high-sensitive flexible tactile sensors have potential application in the field of e-skin, such as monitoring human physical health and simulating human touch perception. Notably, although the transmission mechanisms of discussed works above differ, the flexible tactile sensors all show a high sensitivity and have potential application prospects in the future of intelligent microsystems.

### 3.3. Self-Powered Tactile Sensing

**Figure 4:**
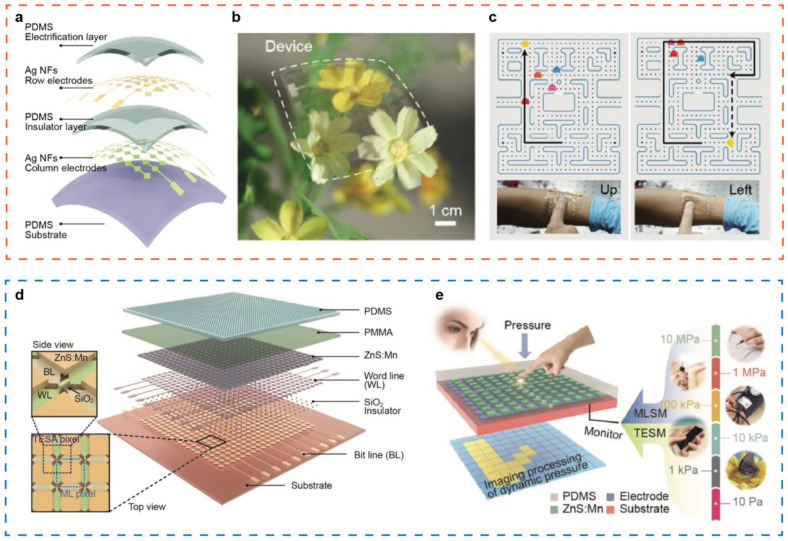
Self-powered flexible tactile sensor. (**a**) The structure diagram of the transparent tactile sensor array. (**b**) A physical image of the tactile sensor. (**c**) Motion detection of the tactile sensing array. Reproduced with permission from Ref. [98]. Copyright 2018, Wiley-VCH. (**d**) Full-range tactile sensor array based on electrical and optical dual mode. (**e**) Illustration diagram of different pressure mapping process and their corresponding potential application. Reproduced with permission from Ref. [102]. Copyright 2017, Wiley-VCH.

Self-powered tactile sensors can directly convert mechanical energy into electrical signals easy to detect without an external power source [100]. TENG based on the triboelectric effect has been widely applied in self-powered flexible tactile sensing. Specifically, the working principles of TENG-based self-powered tactile sensing mainly include contact-separation modes, single electrode modes, and dual modes [79,101]. The TENG based on the contact-separation mode can perform static and dynamic pressure measurement at the same time. In order to obtain real-time local pressure distribution imaging, single-electrode mode TENG has been reported for flexible tactile sensing, which can directly detect the contact behavior of human fingers. The Pan group reported a self-powered single-electrode triboelectric tactile sensor composed of highly stretchable PDMS and patterned silver nanowires, in which the Ag nanofiber electrodes were synthesized by electrospinning and magnetron sputtering [98]. The resistance and the transmittance of the Ag electrodes is 1.68–11.1 Ω^−1^ and >70%, respectively. The orientation of Ag nanofibers is pointed out to be critical to the stretchability. Randomly oriented Ag nanofibers are used to prepare an 8 × 8 cross-type triboelectric sensor matrix. Figure 4a,b show the structure diagram and physical image of the transparent tactile sensor array, respectively. The induced current will be generated in the electrode when the PDMS and the finger contact and separate. Figure 4c display the application design in fast and reliable real-time tactile mapping image and finger motion detection, providing a great platform for touch sensors with irregular planes, which shows a great potential application prospect in touch pad, robots, and wearable electronics.

To obtain more sensitive and wide-range tactile detection imaging, dual-mode TENG has also been investigated extensively. On the basis of the combination of piezoelectric and triboelectric effect, Wang et al. designed and fabricated a self-powered full-range tactile sensor array with a resolution of 100 dpi, basically covering the entire pressure range in our daily life, as shown in Figure 4d [102]. The pressure sensitivity and measurement range can be adjusted by modified PDMS surface. The pressure sensitivity is 6 MPa^−1^ in the range from 0.6 KPa to 200 KPa and 0.037 MPa^−1^ from 650 KPa to 30 MPa. The working process and potential application field of the tactile sensor in different pressure ranges is presented in Figure 4e. In the low pressure range, the triboelectric sensor matrix plays a significant role on acquiring pressure mapping. When a larger pressure applied, mechanoluminescent sensor matrix shows a better sensitivity to the pressure change although both optical and electrical signals can be collected simultaneously with no external power applied.

### 3.4. Visual Tactile Sensing

**Figure 5:**
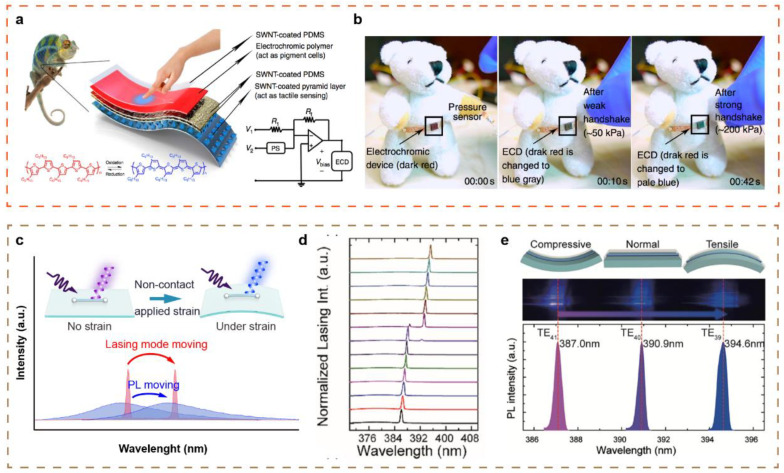
Visual flexible tactile sensing. (**a**) The structural illustration of a chameleon-inspired stretchable electronic skin with color change influenced by tactile perception. (**b**) The expression of tactile sensing into visible color changes. Reproduced with permission from Ref. [103]. Copyright 2015, Springer Nature. (**c**) Schematic diagram of the dynamical modulation of piezoelectric materials lasing mode for strain sensor. (**d**) Lasing spectra shift under different external strain in ZnO microwire. (**e**) The corresponding lasing optical images under compressive and tensile strain. Reproduced with permission from Refs. [104,105]. Copyright 2019, Wiley-VCH. Copyright 2019, Elsevier Ltd.

It has great potential application in the future visual flexible tactile sensing to distinguish pressure value by the change of sensor color. Inspired by the ability of chameleon to change skin color, the Bao group has developed a bionic stretchable electronic skin with color interactive change and tactile sensing characteristics, which is realized by integrating organic electrochromic devices and a resistance-adjustable pressure sensor (Figure 5a) [103]. A pyramidal-microstructured PDMS spray-coated by single-wall carbon nanotubes layer was chosen as the tunable resistive pressure sensor. A P3HT-based organic electrochromic device is prepared on an elastic PDMS substrate. Figure 5b demonstrates the process of expressing tactile information through distinguishable color change. When pressure is applied (less than 50 KPa), the color of the device changes from dark red to blue gray. When the pressure is greater than 200 KPa, the color changes to light blue, and when the pressure is released, the color returns to dark red. The system is expected to be widely used in wearable devices, intelligent robots and other fields.

Subsequently, a series of flexible color-perception non-contact tactile sensors monitoring pressure magnitude via color change have been reported [41,42,43,105,106]. High quality piezoelectric microwire materials were directionally transferred to PET substrates to fabricate the flexible tactile sensor sensing strain change by lasing mode shift, exhibiting significant merits including high color-resolvability, noncontact interactions, high resolution, and a simple construction [104] (Figure 5c), When a strain is applied to the microwires, an obvious shift will be occurred in both lasing mode and PL peak. Figure 5d,e present the lasing spectra and corresponding optical images of ZnO microwires under tensile and compressive strain [105]. The mechanism of strain dynamically modulating lasing mode can be attributed to the strain-induced piezo-polarization effect in piezoelectric materials results in the change of microcavity refractive index. So far, ZnO, GaN and perovskite have been successively researched to prepare the visual flexible tactile sensing [43,104]. The visual flexible tactile sensor has the characteristics of high resolution, being non-contact, having high stability, and being light weight and transparent.

### 3.5. Other Performances of Tactile Sensing

Inspired by the ability of human skin to heal and metabolize, an increasing amount of attention has been concentrated on self-healing, biodegradable materials, and flexible tactile sensors. The characteristic of self-healing can greatly extend the service life of devices when they are damaged. The Bao Group reported the first self-healing tactile sensor, which was fabricated with a supramolecular organic polymer to form a hydrogen bonding network embedded with nickel microparticles [107]. Wang et al. introduced dynamic covalent imine bonds as reversible healing sites into a PDMS network [108]. Moreover, in order to avoid the patient’s second surgery and achieve the goal of safer and simpler treatment of the disease, good biodegradability has also attracted a lot of research. Among them, silicon-based electronic devices play a representative role due to their hydrolysis ability under physiological conditions. Additionally, Wang and co-workers developed a biodegradable TENG to treat heart disease via natural materials, including cellulose, silk fibroin, egg white, rice paper, and chitin [109]. These research results lay a foundation and open up ideas for the further development of high-performance multifunctional flexible tactile sensing.

## 4. Applications for Intelligent Systems

Flexible tactile sensors play a significant role on the development progress of intelligent microsystem since their superior characteristics. Tactile sensing is a cross-domain technology, which can be applied in different application domains (cross-domains). In this section, we mainly introduce the application in the intelligent fields, including wearable electronics used for human health monitoring, intelligent robots used for environmental and object perception, human-computer interaction interfaces for prosthetics and electronic signatures, and implantable electronics for biomedical therapies.

### 4.1. Wearable Electronics

**Figure 6:**
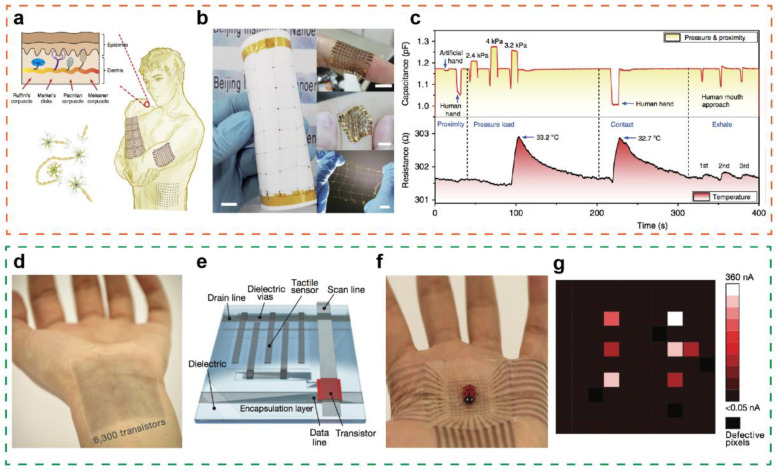
(**a**) Illustration diagram of the sensing system for human skin. (**b**) Optical image of the highly flexible multifunctional sensor network. (**c**) Simultaneous pressure, proximity and temperature stimuli sensing performances. Reproduced with permission from Ref. [113]. Copyright 2018, Springer Nature. (**d**) A large-scale wearable electronic device containing 6300 transistors attached on human twist. (**e**) Single transistor structure based on piezoresistive tactile sensor. (**f**) An artificial ladybug on the skin electronics and (**g**) the corresponding pressure position distribution. Reproduced with permission from Ref. [114]. Copyright 2018, Springer Nature.

With the popularity of intelligent terminals, wearable electronics present a huge market prospect. As one of its core components, tactile sensors will affect the functional design and future development of wearable devices. For wearable electronic products, tactile sensors are the key to realize intelligent sensing function and play a vital role in human health monitoring [83]. Two common approaches, elastic substrate and island-bridge structure, can be employed to realize flexible/stretchable electronic skin [110,111,112]. At present, wearable electronics not only possess tactile sensing characteristics, but also have many practical and potential applications, such as for human temperature, pulse, and motion monitoring. Inspired by human skin, The Wang group and the Pan group have developed a highly flexible multifunctional sensor network with metal island-bridge structure, which can detect seven kinds of signals including temperature, strain, humidity, light, magnetic, pressure and proximity [113]. Figure 6a,b illustrate the sensing system of human skin and the prepared super flexible multifunctional sensor, respectively. Due to the fact that the sensor network system can sense temperature, pressure, and proximity stimuli simultaneously, it has been integrated onto the intelligent prosthesis, which can acquire the pressure distribution and estimate the temperature of the grasped object (Figure 6c). The Bao group subsequently reported a polymer transistor array that can seamlessly adhere to human skin and is comfortable to wear [114]. It contains 347 transistors per square centimeter. (Figure 6d) The structure of a single resistive tactile sensor transistor based on carbon nanotube electrodes is shown in Figure 6e, including tactile sensors, as well as analog and digital circuit elements. The electronic device can be closely attached to the palm of a human hand, and the specific position of the artificial ladybug can be accurately detected by tactile sensing via the current value change (Figure 6f,g). Furthermore, a large-area active tactile sensor matrix based on two-dimensional materials has also been researched, which can be tightly attached to the skin for application in wearable electronics [53]. In addition, tactile sensor arrays based on other working mechanisms including triboelectric, piezoelectric, and pyroelectric effects have also been developed into wearable electronic devices with different functional characteristics. [14,115]

### 4.2. Intelligent Robotics

**Figure 7:**
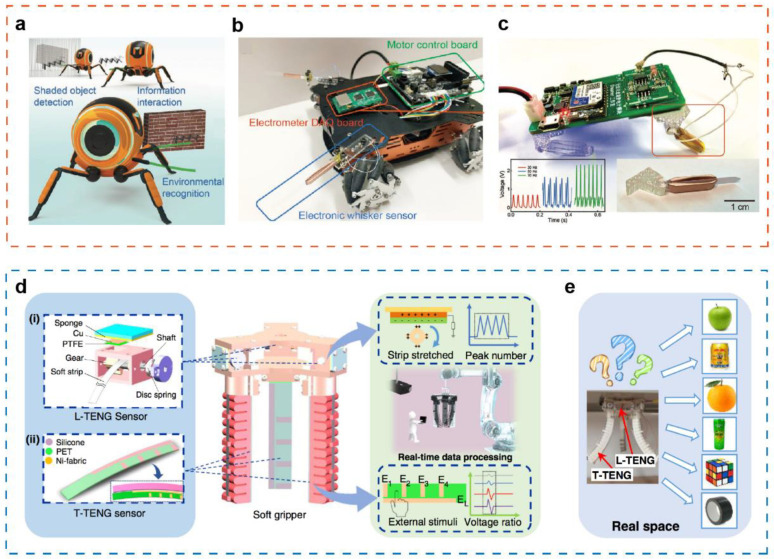
(**a**) Application scenario of a flexible biomimetic whisker sensor. (**b**) An automated guided vehicle equipped with the biomimetic whisker sensor showing applications in shaded object detection and object surface morphology acquisition. (**c**) The quadruped robot assembled by the fabricated system showing the application of vibration detection and road condition perception in robots. Reproduced with permission from Ref. [118]. Copyright 2021, Wiley-VCH. (**d**) Construction and data processing illustration of the TENG-based soft robot gripper. (**e**) The intelligent gripper with capability of perceiving and recognizing various objects. Reproduced with permission from Ref. [119]. Copyright 2020, Springer Nature.

With the development of information technology, a variety of service facilities related to daily life, such as mobile phones, computers, household appliances, as well as media carriers of entertainment and education, have begun the “tactile revolution” [45,116,117]. Tactile sensors are the core device for realizing intelligent robot perception. A flexible biomimetic whisker sensor is designed to construct the tactile sensing system of robot, imitating the way that animals use hair sensors to explore the environment [118] (Figure 7a). The hair follicles of biomimetic whisker mechanoreceptors can sense weak signals, whose sensitivity to external stimuli is as high as 1.129 μN. The tactile sensing system can be installed in different parts of the robot, and plays an important function in robot environment recognition, object surface morphology acquisition, detection of surrounding objects and ground environment, and self-gait analysis (Figure 7b,c). Finally, after the information processing technology such as artificial intelligence and machine recognition, the robot has extraordinary navigation ability and strong environmental adaptability.

In order to improve the robotic abilities to detect contact separation, sliding and continuous motion, further research have been carried out. Especially for soft robots, since their shape is easily deformed under the external pressure, monitoring proprioception is particularly challenging [119,120]. In addition, due to the elastic bodies of soft robots, they often have the characteristics of nonlinearity, hysteresis, and viscoelasticity, making it more difficult to monitor proprioception. Recently, an intelligent soft robot gripper system based on TENG for capturing continuous motion and tactile information has been reported, as shown in Figure 7d [119]. The TENG sensor is composed of two parts: the tactile sensor with pattern electrode and the length sensor with gear structure, which can not only detect the sliding, contact position, and clamping mode, but also measure the bending angle of the soft actuator. Subsequently, two TENG sensors are integrated into the soft robot gripper system for application testing. The intelligent gripper can successfully perceive and recognize various objects with an accuracy of 97.1%, and can be further improved to 98.1% by enhancing the number of sensor channels from 6 to 15 (Figure 7e). Through the intelligent improvement and training to the soft gripper, the intelligent robotic system is expected to be applied to the production control management line in next-generation intelligent factory and the workshop management of the unmanned warehouse. Additionally, the flexible tactile sensor based on the composite ZnO piezoelectric thin film transistor array is prepared, which has good sensitivity to normal force and shear force. The tactile sensor array can be combined with the robot claw to form a closed-loop control system, successfully achieving the clamping and lifting of fragile objects.

### 4.3. Human-Machine Interface

**Figure 8:**
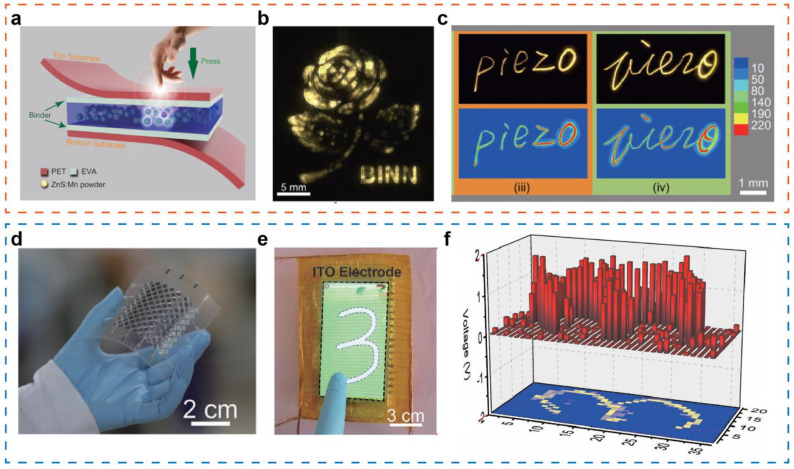
(**a**) A flexible pressure sensor matrix based on mechanoluminescence from ZnS:Mn particles. (**b**) 2D planar pressure distribution through pressing a “piezo” seal on the pressure sensor. (**c**) Handwritten signatures “piezo” and corresponding illumination intensity mapping. Reproduced with permission from Ref. [97]. Copyright 2021, Wiley-VCH. (**d**) Photograph of a cross-type triboelectric sensor array. (**e**) Demonstration of a touching trail “3” on the cross-type triboelectric sensor matrix. (**f**) The pressure distribution mapping corresponding to the touch signature. Reproduced with permission from Ref. [99]. Copyright 2016, Wiley-VCH.

The human-machine interface, as a communication window between the users and specific devices, robot or virtual world, represents a key element to achieve effective, intuitive, and seamless operation to complete given tasks. Wang et al. fabricated a flexible pressure sensor matrix based on mechanoluminescence from ZnS:Mn particles, realizing 2D planar pressure mapping and single point dynamic pressure recording ranging from 0.6-50 MPa [97] (Figure 8a). The 2D planar pressure distribution is shown in Figure 8b, when a rose-shape stamp presses on the device. A signature collection system that can record handwritten signatures and signature habits is built. Figure 8c presents the handwritten signatures “piezo” and corresponding illumination intensity mapping, distinguishing the difference of signatures via morphology, writing pressure, and writing speed. The pressure sensing device could collect more unique and reliable personalized information in the signature process, providing a new way to achieve high-level security.

Moreover, a self-powered high-resolution flexible tactile sensor matrix based on single-electrode TENG has been investigated to accomplish real-time display of touch action or tracking of movement trajectory [99] (Figure 8d). In order to overcome the challenge of a larger number of addressing lines (m × n) to achieve fast mapping when the scale of the sensor matrix is further expanded, this work has developed a new type of interleaved triboelectric sensor matrix that reduces the number of scanning channels to m + n. The cross-type triboelectric sensor matrix has the characteristics of low power consumption and fast addressing capability, which is very suitable for building large-scale and flexible electronic skins for prostheses, robots, and human-machine interfaces. Figure 8e,f shows the pressure distribution mapping corresponding to the touch signature on the device surface when wearing nitrile rubber gloves, which lays the foundation for the extensive application of real-time tactile mapping in human-computer interfaces such as touch sensing and motion tracking. In addition, a smart prosthetic tactile sensor based on a giant magneto-impedance material embedded in an air gap has also been reported, with characteristics including high sensitivity, extremely low detection limit, and digital frequency signal conversion [121]. It has great application potential for intelligent prostheses and artificial interactive interfaces.

### 4.4. Implantable Electronics

**Figure 9:**
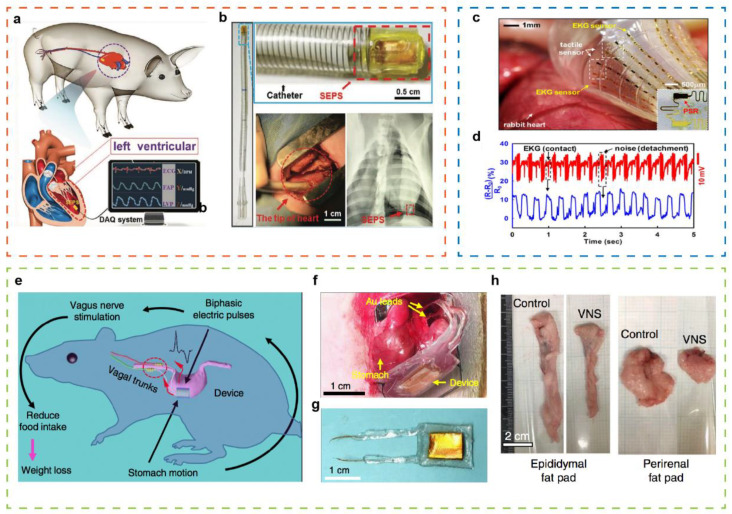
(**a**) Schematic diagram of a flexible TENG implanted into a pig’s heart. (**b**) Optical photograph the whole implantable device. Reproduced with permission from Ref. [123]. Copyright 2019, Wiley-VCH. (**c**) Multifunctional balloon catheters implanted in rabbit heart to acquire cardiac electrophysiological, tactile and temperature signals. (**d**) Electrical activation and mechanical contact recording from the surface of beating heart. Reproduced with permission from Ref. [16]. Copyright 2011, Springer Nature. (**e**) Schematic diagram of an implantable vagus nerve stimulation system. (**f**) Implanted device onto stomach of a rat. (**g**) Photograph of a packaged device. (**h**) Representative images of white adipose tissue of the control and vagus nerve stimulation group. Reproduced with permission from Ref. [124]. Copyright 2020, Springer Nature. Copyright 2018, Springer Nature.

With the rapid development of electronic technology and biomedicine, implantable electronics are more and more widely used in clinical research. Implantable electronics can monitor physiological signals in time and even achieve therapeutic effects. Tactile sensors based on TENG attach an increasing number of researchers’ attention to biomedical applications in vivo, since it has advantages including sensing, powering, and biosafety [122]. A miniature, flexible, and self-powered endocardial tactile sensor based on TENG is investigated, integrated with surgical catheter for minimally invasive implantation to detect endocardial pressure [123] (Figure 9a,b). 

Then, the sensors were implanted into the left ventricle and left atrium of a pig, possessing a sensitive real-time response and mechanical stability in both low and high-pressure environments. In addition, the endocardial pressure sensor can detect arrhythmias, such as ventricular fibrillation and premature ventricular contractions. The device may promote the development of micro implantable medical sensors for monitoring and diagnosing cardiovascular diseases, which has an extensive application prospect in the implanted health supervision field. Moreover, a multifunctional sensor can be utilized to test tactile, temperature, flow, optical, electrophysiological data and to control local ablation of tissue, which has also been implanted into rabbit heart for cardiac ablation therapy [16] (Figure 9c,d).

A flexible and biocompatible nanogenerator attached to the surface of the stomach was also demonstrated, forming an implantable vagus nerve stimulation system, as displayed in Figure 9e–g [124]. The electrical signal generated by the device can stimulate the afferent fibers of the vagus nerve, thereby reducing food intake and achieving weight control. When implanting the device into the rat model, the average weight of rat was 350 g within 100 days (38% less than the control group), which provides a method to realize therapeutic purpose through self-responsive and neuro modulation (Figure 9h). Along with the further development of implantable electronic devices, biodegradable component materials have been gradually studied and employed in the transient electronics and physiological monitoring.

## 5. Conclusions and Perspectives

In this review, recent advances on flexible tactile sensors, from working mechanism to performance and from design to application, are introduced. Transmission mechanisms mainly including piezoresistivity, capacitance, piezoelectricity, and triboelectricity and their corresponding materials selection and characteristics are summarized, respectively. The improvement of resolution, sensitivity, and other performance in flexible tactile sensors has been presented. At the same time, flexible tactile sensors are developing towards the multifunctional direction. Smart flexible sensors with self-power, visualization, biodegradable, and other practical functions have been studied, which broadens the application field of intelligent systems and enhances user experience. It will promote the further development of wearable devices, intelligent robots, human-computer interaction systems, and biomedical systems.

Although many breakthroughs have been made in the field of flexible tactile sensing in recent years, there are still many challenges faced in practical applications such as the performance degradation of the sensor during repeated deformation, the crosstalk decoupling of multi-dimensional and multi-stimulus simultaneous detection, the mechanical, thermal, and electrical performance matching among the internal components of the integrated sensing system, etc. These challenges bring new development opportunities, and point out the future development direction for related material preparation, device processing, and system integration. Specifically, first of all, the device will undergo repeated deformation in the actual use process, leading to performance degradation. Therefore, more efforts can be made to enhance the robustness and prolong the service life of the device in the future. The researches on advanced materials and structures and the introduction of advanced packaging technology are the technical development directions. Secondly, crosstalk is an important problem that influences the simultaneous acquisition of multiple signals. How to decouple crosstalk signals greatly affects its application in intelligent systems. One of the effective methods is using transistor structure to reduce crosstalk. In addition, the mechanical, thermal, electrical, and optical properties of each component in the integrated sensing system should be matched. There is no doubt that the tactile sensor will develop in the direction of being more flexible, miniaturized, intelligent, multifunctional and humanized. Its applicable boundary will also be greatly widened, playing a more irreplaceable role in more intelligent fields.

Flexible tactile sensors are a cross-cutting frontier research field that integrates flexible electronics, device physics, and materials science, which have great application potential in health monitoring, flexible touch screens, flexible electronic skin, medical diagnosis, virtual electronics, and even industrial robots. One promising way to improve the dexterity and adaptability of robots is to provide them with tactile sensations. In response to this, electric skins supported by the advancement of various tactile sensors have been proposed to allow robots to operate safely and accurately in an unknown, uncertain, and cluttered environment. Artificial intelligence is among the primary driving forces for the development of tactile sensors, in which tactile sensors endow cognitive functions and then learning capability to machines. However, the integration of tactile sensors in artificial intelligence is still in its infancy due to the immaturity of coupling signals to terminals, a function similar to nerves in human, which is a significant research direction. The future trends of flexible tactile sensors will develop towards multi-function, integration, and intelligence. With the further development of materials science, flexible electronics and nanotechnology, the performance and function of tactile sensors will improve rapidly. Combined with the advanced intelligent feedback haptic, flexible tactile sensors with precise sensing accuracy and high reliability have great application value in the field of intelligent systems.

## Figures and Tables

**Table 1:** Summary of some flexible tactile sensors and their performance indicators.

Transduction Mechanisms	Materials and Structure	Sensitivity	Resolution	Response Time	Reference
piezoresistive	Crack Pt/PUA	2000	-	-	[92]
piezoresistive	PDMS/SWNTs	1.8 kPa^−1^	-	<10 ms	[93]
piezoresistive	Polypyrrole	133.1 kPa^−1^	-	50 ms	[94]
piezoresistive	PDMS/Ag	44,013	-	87 ms	[58]
capacitive	Pyramid-structured PDMS	0.55 kPa^−1^	-	millisecond	[33]
capacitive	PDMS/carbon nanotubes	-	2 mm	≤125 ms.	[66]
capacitive	PDMS/air gap	0.7 kPa^−1^	-	-	[95]
capacitive	GNPs/MWCNTs/SR/PS 3D Porous composite	0.062 kPa^−1^	-	∼45 ms	[20]
piezoelectirc	GaN/ZnO NWs	12.88 GPa^−1^	6350 dpi	90 ms	[9]
piezoelectirc	Flexible GaN/ZnO NWs	-	2.6 μm	180 ms	[88]
piezoelectirc	PET/ZnO NWs/PEDOT:PSS	-	7 μm	-	[72]
piezoelectirc	ZnO nanoplatelet	60.97–78.23 meV MPa^−1^	12,700 dpi	<5 ms	[89]
piezoelectirc	PI/ZnO TFTs	-	100 μm	<10 ms	[27]
Triboelectric	Wrinkled PDMS/MXene	0.18 V/Pa	-	-	[96]
Triboelectric	PET/ZnS:Mn particles	2.2 cps/KPa^−1^	<100 µm	10 ms	[97]
Triboelectric	PDMS/Ag nanofibers	-	-	70 ms	[98]
Triboelectric	PDMS/Al	0.06 kPa^−1^	2.5 mm	70 ms	[99]

## Data Availability

Not applicable.
